# The New Frontiers – Creating a “Thyroid Home”

**DOI:** 10.3389/fendo.2015.00047

**Published:** 2015-03-30

**Authors:** Terry Francis Davies

**Affiliations:** ^1^Department of Endocrinology, Diabetes and Bone Diseases, Icahn School of Medicine at Mount Sinai, New York, NY, USA

**Keywords:** thyroid endocrinology, editorial, thyroid physiology, thyroid pathology, thyroidology

It is 2015. There are plenty of good things happening within the thyroid community. There are also plenty of disappointing things happening within the thyroid community. Such is life. Maybe as a first Editorial for Thyroid Endocrinology, a section of the journal Frontiers in Endocrinology, we should take a look at the current scene.

As far as the good things are concerned, we should be celebrating the amazing technical advances in almost all areas of laboratory research, which are allowing us to ask important new questions. Disappointingly, the cost of such research has risen to astronomical amounts and the funding available, at least in the USA and I suspect almost everywhere, is falling more and more behind the costs. The late Sydney Ingbar was able to keep an entire thyroid unit funded with one NIH grant. Today, you need three grants just to get some quality work done.

We should also be celebrating how much thyroid physiology and pathology are involved in new areas – far away from anywhere we expected – such as the environment, the brain, bone repair, and the immune system giving new opportunities for knowledge and funding. Unfortunately, not many thyroidologists have moved to take up these opportunities and when they do the entrenched members of the brain and bone and immune communities resent the invasion. Our societies and NIH need to issue some calls for research in these areas.

Thyroid cancer clinicians should be celebrating the rising tide of kinase inhibitors able to control thyroid cancers. But the patients are not celebrating yet – they are waiting for the good life to be significantly extended more than a few months. With the advent of check point inhibitors, even better results may be around the corner.

And in Europe, we should be celebrating the fact that thyroidology continues to attract bright young investigators to follow their heart and develop a career in clinical or laboratory research. Disappointingly, this is not so in the USA where there remains a dearth of home grown young thyroid investigators because of the lack of stability in funding and the widespread but short sighted intention to appoint diabetologists to every position possible. A strategy to correct this deficit needs to be formulated by our Professional Societies. The availability of starter grants provides no security with their annual renewal requirements and are so often written by the senior mentor that it has become a joke after reading highly sophisticated applications written by first year clinical fellows. Rather, we need to support more Research Fellowships allowing young investigators to become established and generate new data for future grant applications. And the salaries for such Fellowships need to be attractive. In fact, I would probably recommend abandoning the whole idea of starter grants and spend the money investing directly in the most able young people and the most able mentors. Obviously, there would need to be some agreed research plan but only a broad outline including training opportunities.

We should also be celebrating the ease with which it is now possible to publish our work. No longer is there a stranglehold by a small elite. The multiplicity of outlets both in print and more importantly on the internet has allowed the dissemination of much more work and with much more speed. Disappointingly, this has clearly resulted in a lowering of quality barriers to publication. The proliferation of online research journals has been astonishing and the evidence that many provide only cursory reviews or none at all is astounding! Frontiers stands out from the crowd in this regard. We have an excellent interactive review system and our new Board of Associate Editors is one I am most proud of. This is our assurance of future quality publications. I invite you to contribute. Whether it be clinical or bench research, opinion, or illustrations, this is a vital Thyroid Home you can contribute to, enjoy, and learn from. Come aboard!

## Conflict of Interest Statement

The author declares that the research was conducted in the absence of any commercial or financial relationships that could be construed as a potential conflict of interest.

